# Green synthesis of silver nanoparticles derived from lemon and pomegranate peel extracts to combat multidrug-resistant bacterial isolates

**DOI:** 10.1186/s43141-023-00547-0

**Published:** 2023-10-10

**Authors:** Mohamad Abdelrazik, Hassan H. Elkotaby, Abdullah Yousef, Ahmed F. El-Sayed, Mohamed Khedr

**Affiliations:** 1 Botany and Microbiology Department, Faculty of Science, Suez Canal University, Ismailia, Egypt; 2 Department of Botany and Microbiology, Faculty of Science, Al-Azhar University, Nasr, Cairo, 11884 Egypt; 3 Microbial Genetics, Biotechnology Research Institute, National Research Centre, Giza, Egypt; 4 Egypt Center for Research and Regenerative Medicine (ECRRM), Cairo, Egypt

**Keywords:** Lemon peel extract, Pomegranate peel extract, Silver nanoparticles, ISSR, SDS-PAGE, Multidrug-resistant bacteria

## Abstract

**Background:**

Multidrug-resistant (MDR) bacteria are acknowledged as one of the main factors contributing to chronic illnesses and fatalities globally. Numerous diseases, including bloodstream infections, pneumonia, urinary tract infections, and surgical site infections, can be brought on by MDR bacteria. Therefore, a crucial topic of continuing research is the development of a novel and different treatment for MDR microbial pathogens. This work is introduce an alternative method for elimination of MDR bacterial isolates which are causative agents of urinary tract infection among people in Egypt. In our study, we need a novel strategy to combat MDR bacteria by green-synthesized metal nanoparticles (MNPs). That is due to the ability of MNPs to penetrate the cell wall and the cell membrane of gram-positive and gram-negative bacteria.

**Methods:**

Clinical isolates of MDR bacteria had their antibiotic susceptibility assessed before being molecularly identified using 16 s rRNA, sequencing, and phylogenetic analysis. Also, genetic profiles of isolated strains were performed using ISSR and SDS-PAGE. Finally, characterized plant-mediated silver nanoparticles derived from lemon and pomegranate peel extracts were evaluated against isolated multidrug-resistant bacterial stains.

**Results:**

In our present trial, one-hundred urine samples were collected from 71 females and 29 males complaining of UTI (urinary tract infection) symptoms. One-hundred microbial isolates were isolated, including 88-g negative and only 8-g positive bacteria in addition to four yeast isolates (*Candida* species). A total of 72% of the isolated bacteria showed MDR activity. The most prevalent MDR bacterial isolates (*Escherichia coli*, *Pseudomonas aeruginosa*, *Acinetobacter baumannii*, *Enterococcus faecalis*, and *Klebsiella pneumoniae*) were identified through 16S rDNA PCR sequencing as with accession numbers OP741103, OP741104, OP741105, OP741106, and OP741107, respectively. Lemon and pomegranate-mediated silver nanoparticles [Ag-NPs] were characterized by UV spectroscopy, FTIR, XRD, and TEM with average size 32 and 28 nm, respectively. Lemon and pomegranate-mediated silver nanoparticles [Ag-NPs] showed an inhibitory effect on the selected five MDR isolates at MIC 50 and 30 µg/mL, respectively. These common bacterial isolates were also genetically examined using ISSR PCR, and their total protein level was evaluated using SDS-PAGE, showing the presence of distinct genetic and protein bands for each bacterial species and emphasizing their general and protein composition as a crucial and essential tool in understanding and overcoming MDR behavior in UTI patients.

**Conclusions:**

Lemon and pomegranate-mediated silver nanoparticles [Ag-NPs] were found to have an inhibitory effect on MDR isolates. Therefore, the study suggests that [Ag-NPs] could be a potential treatment for MDR UTI infections caused by the identified bacterial species.

**Supplementary Information:**

The online version contains supplementary material available at 10.1186/s43141-023-00547-0.

## Background

UTI (bacteriuria) is the medical term for the presence of bacteria in the urine. Although clinical infection might start at 103 bacteria/mL, “remarkable” bacteriuria for epidemiological purposes should be at least 105 bacteria/mL in freshly voided urine [1]. UTI is the most frequent infectious illness in the world, impacting more than 10% of the world’s 150 million individuals per day. Clusters of virulence factors are a significant particular cause of multidrug resistance in the urinary tract [2]. MDR pathogens in urine make UTI harder to treat empirically and increase infection prevalence [3–5]. Worldwide, the uncommon appearance and growth of multidrug-resistant bacterial infections are a serious public health risk that is emerging in ever-expanding communities [6, 7]. Over the past few decades, the emergence of MDR uropathogens has led to an increase in hospital-acquired UTI infections worldwide [8, 9]. Many microbial species and isolates have not yet been identified or described [10]. Several identification techniques are currently available, ranging from total protein and isozyme profiles to DNA/DNA hybridization and sequence analysis of the 16S rDNA region [11–13]. The availability of molecular markers such as 16S rDNA [14], RAPD [15, 16], and ISSR [17] facilitates the development of phylogenetic relationships and the identification and characterization of bacterial species. Protein banding patterns using sodium dodecyl sulfate–polyacrylamide gel electrophoresis (SDS-PAGE) technique are used to distinguish infectious wild-type organisms from their mutants [18]. The biosynthesis of metal oxide nanoparticles utilizing biosynthetic agents derived from plants or microbes is best suited for producing nanomaterials that are already surrounded by cellular proteins, eliminating the requirement for additional cell outer layers or polymers. It is one of the potential approaches [19]. Metal oxide nanoparticles, such as silver, titanium, and zinc oxide nanoparticles, have been extensively researched for their antibacterial effectiveness against different MDR illnesses [20, 21]. The effect of metal oxide nanoparticles in reducing MDR bacteria growth is highlighted by a range of cellular damaging and molecular processes. Metal nanoparticles bind to cellular proteins, altering membrane permeability and protein structure, or to DNA structures, interfering with proper cell division and MDR growth [22]. Third- and fourth-generation cephalosporins and monobactams can be hydrolyzed by extended-spectrum beta-lactamase (ESBL) enzymes, whereas cephamycins and carbapenems cannot. Sulbactam or tazobactam inhibits them, and the genetic determinants are typically associated with plasmids but frequently result in co-resistance (aminoglycosides, quinolones). Due to the high prevalence of *E. coli* in the general population and the fluctuating incidence of *K. pneumoniae* (which is higher in hospitals), ESBLs pose a public health risk [23]. Size, shape, surface functionalization, surface charges, and the capacity to co-encapsulate drugs all affect how effective metal nanoparticles (MNPs) are at fighting multidrug-resistant bacterial infections. Recent research has focused heavily on the effectiveness of silver, gold, zinc oxide, selenium, copper, cobalt, and iron oxide nanoparticles against multidrug-resistant bacterial infections [24].

## Methods

### Isolation of clinical samples

Urine samples were collected from 100 patients (29 males and 71 females), showing symptoms of UTI, attending the outpatient clinics of Suez Canal Authority Hospital during over 8-month period (May 2020–March 2021). The patient’s age ranged from 15 to ≥ 60 years.

### Antibiotic susceptibility

Antibiotic sensitivity test of twenty-five different antibiotics was performed biochemically through disc diffusion method on Muller-Hinton agar (according to guidelines of CLSI 2020). Then, the resistant bacterial isolates were confirmed using VITEK 2 system [25].

### Molecular identification and genetic diversity studies

Genetic diversity was performed for the most prevalent bacterial isolates using intersimple sequence repeat (ISSR), 16 s rRNA, and sequencing, and phylogenetic tree was constructed. DNA extraction from bacterial isolates was performed using the (QIAamp DNA Mini Kit, Qiagen, Germany) according to manual instructions, six ISSR primers were used (1-6) as in Table 1.

**Table 1:** Different six primers used in ISSR amplification of tested five MDR isolates

ISSR1 5-ACACACACACACACACG-3	ISSR2 5-ACACACACACACACACT-3
ISSR3 5-ACACACACACACACACC-3	ISSR4 5-AGAGAGAGAGAGAGAGC-3
ISSR5 5-CTCTCTCTCTCTCTCTG-3	ISSR6 5-CTCTCTCTCTCTCTCTCTA-3

### Data analysis of genetic diversity

The SIMQUAL module was used to determine genetic similarity based on the Jaccard coefficient, and the NTSYS version 2.20 software package was used to tree the data using the unweighted pair-group method with the arithmetic mean (UPGMA) option of the SAHN module. A graph has been made. PAST version 3.16 was used to perform principal coordinate analysis (PCoA) to link genetic relationships between populations (MDR UTI-causing bacteria). Protein profiles were captured as binary data, that is, 1 or 0 depending on the presence or absence of bands, and gels were viewed immediately with the naked eye. A band was indicated as 1 if it was present in a genotype, but it was marked as 0 if it was totally present. The similarity and association between the protein traces of test strains were displayed in a dendrogram using the unweight pair-group method with arithmetic averages algorithm (UPGMA) of the protein patterns of whole-cell protein of species.

### Statistical cluster analysis

Using unweighted pair-group method with average (UPGMA) and principal components analysis (PCA) was performed with PAST 3.18 software [26]. The polymorphic information content (PIC) value was calculated according to the given formula [27]. The percentage polymorphism was calculated according to the following formula. Polymorphism (%) = (number of polymorphic bands/total number of bands) × 100.

### Synthesis of nanoparticles

Lemon peel and pomegranate fruit peel were hand pulverized after being taken from the whole fruit and got dried in an oven until totally dried. About 50-g dried fruit peel powders were suspended to make extracts. For 1 h, the resultant mixtures were sonicated with occasional vortexing. The suspensions were then filtered through Whatman No. 1 filter paper, excess methanol from the filtrate was evaporated using a rotary evaporator, residual water was removed by lyophilization, and the filtrate was stored at 4 °C until further use [28].

#### Silver nanoparticles [Ag-NPs] materials

Fresh lemon leaves and pomegranate peel as reducing agents have been purchased from commercial markets, and the identification was carried out through morphological characters at the Department of Botany, Faculty of Science, Al-Azhar University [28]. Silver nitrate [AgNO_3_] was bought from Sigma-Aldrich with ≥ 99.5% purity from Egypt.

#### Preparation of leaves extract

Fresh lemon leaves and pomegranate peels were obtained from the market and gathered and properly rinsed with tap water before being extensively cleansed with sterile Milli-Q water until removing all contaminants. To get the leaves extract, 5 g of leaves and peels were combined with 100 mL of distilled water and heated for 5 min at 80 °C before chilling and filtering through Whatman No. 1 filter paper. The collected filtered leaf extract was utilized immediately for further research [29, 30].

#### Silver nanoparticles synthesis

A 50-mL solution from 5-mM silver nitrate was produced. Separately, 0.05 g/mL of two extracts was gradually poured on silver nitrate solution at 60 °C to synthesize the colloid. The fast shift in hue to brownish yellow after 5 min indicates that the silver ions formed quickly, validating the creation of Ag-NPs, and there was no further change in color. The Ag-NPs were then moved to a clean container in a dark environment for future research [31, 32].

#### Silver nanoparticles characterization

Characterization of Ag-NPs was accomplished by visual observation and other procedures; in a visual observation, the change in color solution from colorless to reddish brown was noticed with the naked eye indicating the formation of [Ag-NPs]. UV–visible spectroscopy was used to identify Ag-NPs. FTIR spectroscopy was used to study biomolecules in lemon solution as well as the surface chemistry of Ag-NPs. Transmission electron microscopy (TEM) was used to confirm the surface shape and particle size of the Ag-NPs using high-resolution pictures [33, 34].

#### Antibacterial activity of NPs

Both Ag-NPs from two plant extracts were evaluated for their potential bactericidal activity against *Escherichia coli*, *Pseudomonas aeruginosa*, *Acinetobacter baumannii*, *Enterococcus faecalis*, and *Klebsiella pneumoniae* through the well agar diffusion technique on Muller-Hinton medium for 24 h at 37 °C. Fifty liters of NP solution was put within an agar well and then allowed to diffuse before being incubated.

## Results and discussion

### Isolation of bacterial isolates of UTI patients

#### Bacterial species isolated from UTI cases

One-hundred mid-stream urine samples were collected from 71 females and 29 males, attending the outpatient departments (OPDs) (64%) and in-patient departments)36%) of Suez Canal Authority Hospital during over 8-month period (May 2020–March 2021). Clinical evidence of urinary tract infection was determined by urologists. Patient’s age ranged from 10 to ≥ 60 years with average age 58 years. Patients on antibiotic therapy were excluded from the study. Age < 60 years was the most common age group in the study population (51%), followed by the age group of 30–60 years (34%) and < 30 years (15%). The recovered microbial isolates were divided into 88-g negative bacteria (88 isolates), gram positive ones (only eight isolates), and four yeast isolates (*Candida* sp.).

Standing on VITEK report of biochemical analysis of one-hundred isolates was defined as shown in the Table 2.

**Table 2:** Frequency of bacterial isolates of UTI patients

Microorganism	Number (%)
Gram-negative bacteria	
*E. coli*	46 (46%)
*Klebsiella pneumoniae*	24 (24%)
*Pseudomonas aeruginosa*	10 (10%)
*Enterobacter cloacae*	2 (2%)
*Acinetobacter baumannii*	1 (1%)
*Citrobacter koseri*	1 (1%)
*Serratia liquefaciens*	1 (1%)
*Serratia plymuthica*	1 (1%)
*Sphingomonas paucimobilis*	1 (1%)
*Morganella morganii*	1 (1%)
Gram-positive bacteria	
*Staphylococcus lentus*	2 (2%)
*Staphylococcus haemolyticus*	2 (2%)
*Staphylococcus xylosus*	1 (1%)
*Enterococcus faecalis*	1 (1%)
*Staphylococcus saprophyticus*	1 (1%)
*Enterococcus* spp.	1 (1%)
Yeast	
*Candida tropicalis*	3 (3%)
*Candida dubliniensis*	1 (1%)

### Antibiotic susceptibility

All isolates were tested against different groups antibiotics depending on type of genus and species of the tested isolates; after conducting statistical operations, the samples were selected of *E. coli* (*n* = 2), *E. faecalis* (*n* = 21), *P. aeruginosa* (*n* = 38), *A. baumannii* (*n* = 45), and *K. pneumoniae* (*n* = 87) as the most resistant isolates to antibiotics to conduct genetic and protein experiments on them for which their results were as follows in Table 3.

**Table 3:** Antibiogram of selected microorganisms isolated from UTI-infected patients (*n* = 100) against different twenty-five antibiotics

Species	Resistant to (n) (%)	Antibiotics symbols
*E. coli*	15 (83.33%)	AMP, SAM, TZP, FOX, CAZ, CRO, FEP, MEM, AK, TOB, CIP, LEV, SXT, ATM, ETP
*E. faecalis*	5(71.42%)	E, RD, CIP, LZD, TE
*P. aeruginosa*	9 (81.81%)	CAZ, FEP, MEM, CN, TOB, CIP, LEV, ATM, IPM
*A. baumannii*	10 (83.33%)	SAM, TZP, CAZ, CRO, FEP, MEM, CN, TOB, CIP, LEV
*K. pneumoniae*	17 (94.44%)	AMP, SAM, TZP, FOX, CAZ, CRO, MEM, AK, CN, TOB, CIP, LEV, F, SXT, ATM, ETP, IPM

*AMP* ampicillin, *SAM* ampicillin/sulbactam, *TZP* piperacillin/tazobactam, *FOX* cefoxitin, *CAZ* ceftazidime, *CRO* ceftriaxone, *FEP* cefepime, *MEM*, meropenem, *AK* amikacin, *CN* gentamicin, *TOB* tobramycin, *CIP* ciprofloxacin, *F* nitrofurantoin, *SXT* trimethoprim/sulfamethoxazole, *ATM* aztreonam, *ETP* ertapenem, *IPM* imipenem, *E* erythromycin, *MXF* moxifloxacin, *RD* rifampicin, *LZD* linezolid, *DA* clindamycin, *TE* tetracycline, *TEC* teicoplanin, *LEV* levofloxacin

### Genetic diversity of selected isolates using ISSR

**Fig. 1:**
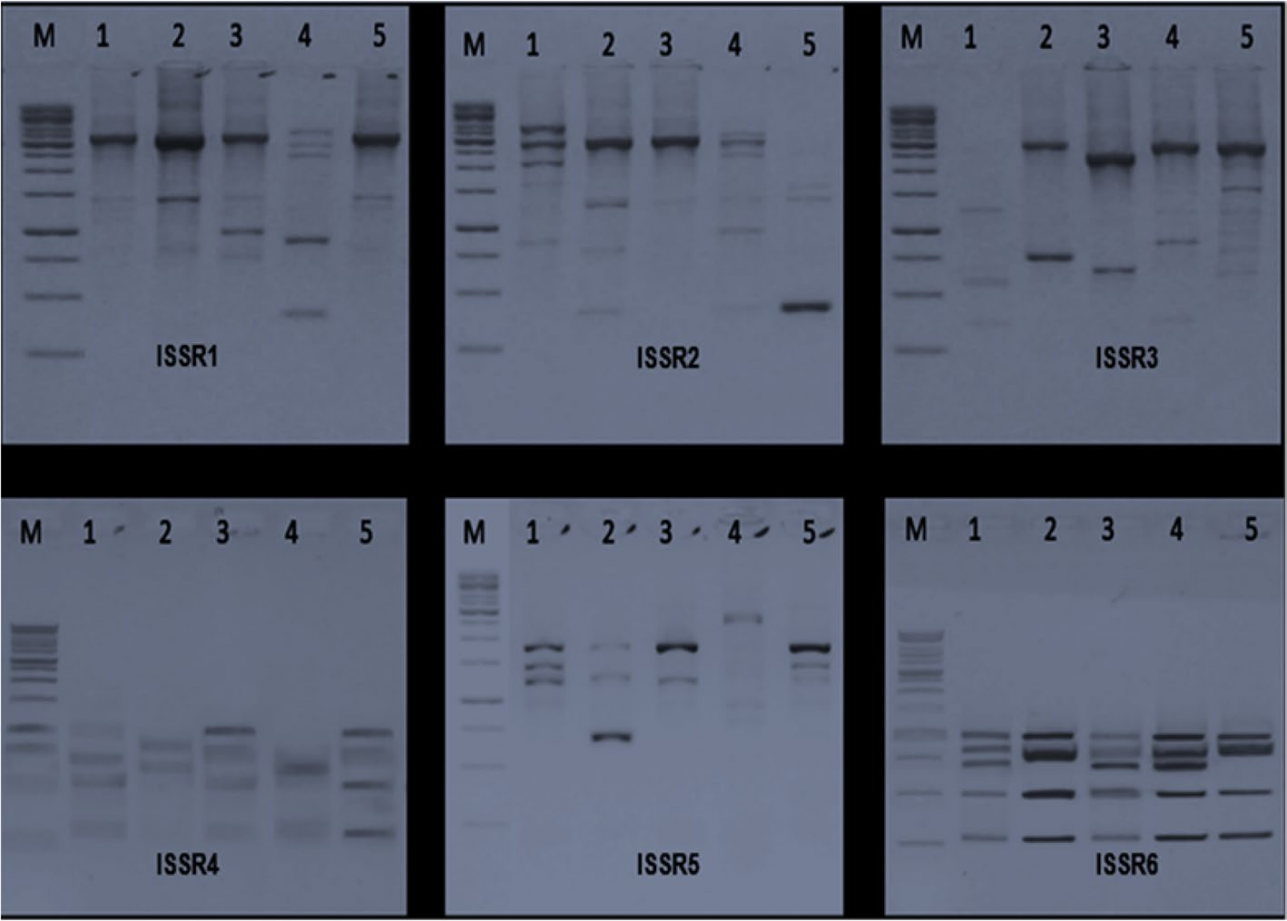
Patterns of ISSR electrophoresis of selected strains. Lane 1: DNA ladder (PageRuler™ Plus Prestained). Lanes 2–6: represent isolates from (1–5) which represent as *E. coli*, *P. aeruginosa*, *A. baumannii*, *E. faecalis*, and *K. pneumoniae*

ISSR-PCR is considered to be an effective approach for phylogenetic categorization of bacterial (prokaryotic) genomes in general, as well as diagnostic genotyping of microbial pathogens. Using six ISSR primers, a DNA sample representing the five bacterial strains was submitted to PCR analysis. SSR analysis can be helpful for figuring out how genotypes relate to population information like morphology, illness susceptibility, and location [35]. The primers were produced a consistent and reproducible banding pattern for the strain tested, which is explain in Fig. 1. According to the data in Table 4, the polymorphism percentage (P%) and polymorphism information content (PIC) for the pattern formed by the ISSR marker revealed that the total DNA fragments produced were 117 fragments with molecular weights ranging from 250 to 2500 bp. The six ISSR primers used for five bacterial pathogens were found to be polymorphic, yielding a total of 36 bands, 16 of which were polymorphic. whereas 13 bands were monomorphic. The polymorphism percentages (P%) of applied six primers were 57.17, 42.85, 60.00, 20.00, 66.66, and 16.66%, respectively. Both primers (3 and 5) have the highest P% with 60.00% polymorphism.

**Table 4:** Polymorphism generated by ISSR of the selected strains

**Primer**	**MW**	***E. coli***	***P. aeruginosa***	***A. baumannii***	***E. faecalis***	***K. pneumoniae***	**Frequency**	**Polymorphism**	**PB**	**MB**	**Unique**	**TB**	**P%**	**PIC**
ISSR1	4000	0	0	0	1	0	**20**	**Unique**						
	3000	1	1	1	1	1	**100**	**Monomorphic**	4	1	2	7	57.17	0.320
	2500	1	1	0	1	0	**60**	**Polymorphic**						
	1500	1	1	1	0	1	**80**	**Polymorphic**						
	750	0	0	1	1	0	**40**	**Polymorphic**						
	500	0	0	0	1	1	**40**	**Polymorphic**						
	250	0	0	0	1	0	**20**	**Unique**						
ISSR2	**3500**	1	0	0	0	1	**40**	**Polymorphic**	4	1	2	7	57.14	0.297
	**3100**	1	0	0	0	1	**40**	**Polymorphic**						
	**3000**	1	1	1	1	1	**100**	**Monomorphic**						
	**1500**	1	0	0	0	0	**20**	**Unique**						
	**1000**	1	0	0	0	0	**20**	**Unique**						
	**750**	0	1	1	1	1	**80**	**Polymorphic**						
	**400**	0	1	0	1	1	**60**	**Polymorphic**						
ISSR3	**3000**	0	1	1	1	1	**80**	**Polymorphic**	3	1	1	5	60.00	0.320
	**1500**	0	0	0	0	1	**20**	**Unique**						
	**1000**	1	0	0	0	1	**40**	**Polymorphic**						
	**750**	1	1	1	1	1	**100**	**Monomorphic**						
	**500**	1	0	0	1	0	**40**	**Polymorphic**						
ISSR4	1000	1	0	1	0	1	**60**	**Polymorphic**	1	4	0	5	20.00	0.0961
	750	1	1	1	1	1	**100**	**Monomorphic**						
	650	1	1	1	1	1	**100**	**Monomorphic**						
	500	1	1	1	1	1	**100**	**Monomorphic**						
	250	1	1	1	1	1	**100**	**Monomorphic**						
ISSR5	2500	0	0	0	1	0	**20**	**Unique**	4	0	3	7	57.14	0.3733
	2200	1	1	1	0	1	**80**	**Polymorphic**						
	2000	1	0	0	0	1	**40**	**Polymorphic**						
	1500	1	1	1	0	1	**80**	**Polymorphic**						
	750	1	0	1	1	1	**40**	**Polymorphic**						
	700	0	0	0	1	0	**20**	**Unique**						
	600	0	1	0	0	0	**20**	**Unique**						
ISSR6	1000	1	1	1	1	1	**100**	**Monomorphic**	1	5	0	6	16.66	0.081
	950	1	1	1	1	1	**100**	**Monomorphic**						
	850	1	0	1	1	0	**60**	**Polymorphic**						
	750	1	1	1	1	1	**100**	**Monomorphic**						
	500	1	1	1	1	1	**100**	**Monomorphic**						
	250	1	1	1	1	1	**100**	**Monomorphic**						
**No. of fragments**		**27**	**20**	**21**	**21**	**28**	**117 fragments**		16	13	7			

*PB* number of polymorphic bands, *MB* number of monomorphic bands, *TB* number of total bands, *%P* percent polymorphism, *PIC* polymorphism information content

**Fig. 2:**
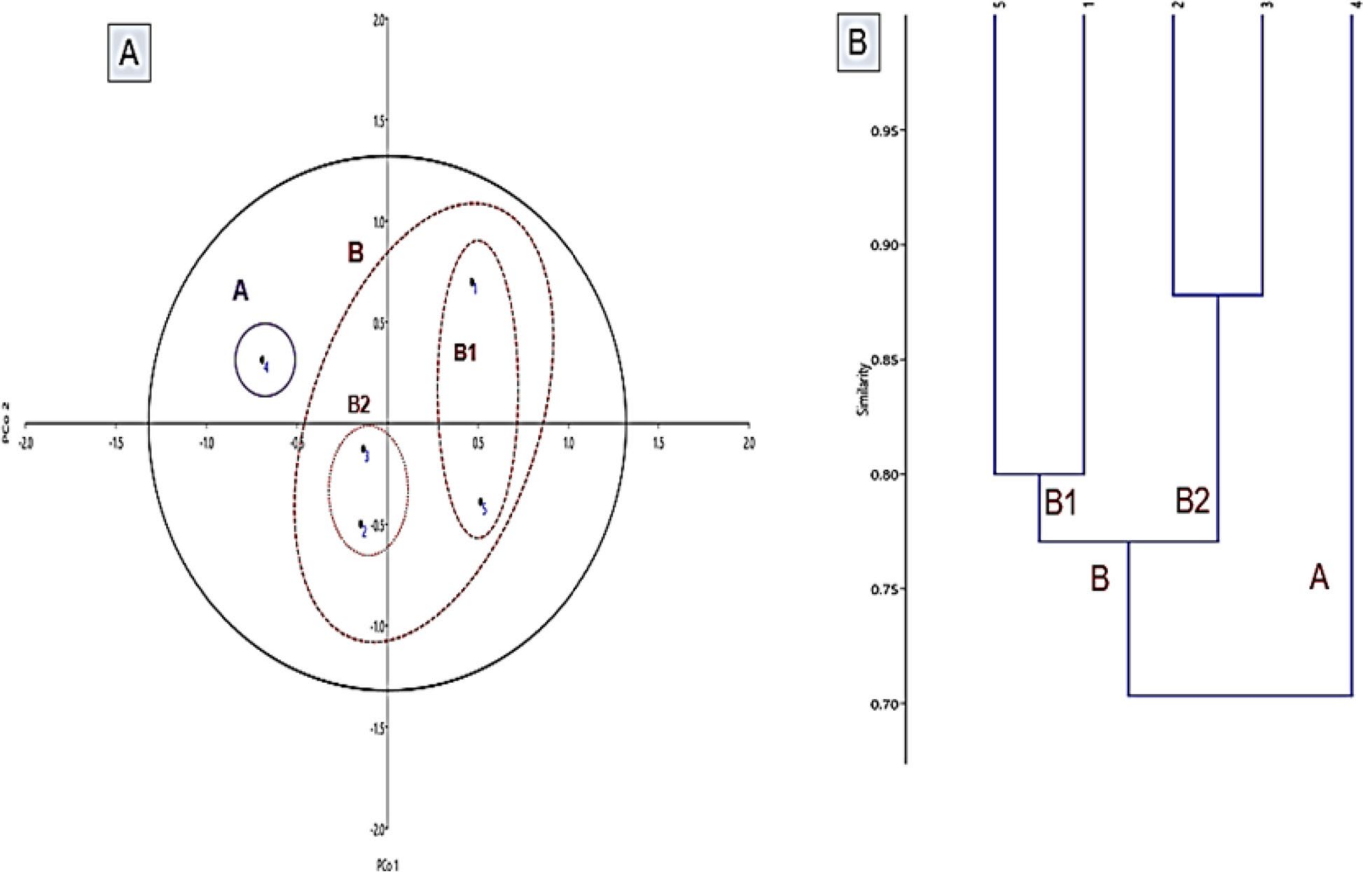
**A** Principal correlation analysis (PcoA) selected strains. **B** Dendrogram of ISSR based on unweighted pair-group method with arithmetic averages algorithm (UPGMA) for selected strains, isolates code (1–5) was represented as *E. coli*, *P. aeruginosa*, *A. baumannii*, *E. faecalis*, and *K. pneumoniae*

Two unique bands produced by three primers (1, 2, and 5) for pathogens are as follows: *E. coli* and *K. pneumoniae*, respectively. Also, polymorphism information contents (PIC) for ISSR primers (1, 2, 3, 4, 5, and 6) were calculated as 0.320, 0.297, 0.320, 0.0961, 0.3733, and 0.081 generated from ISSR pattern. As shown in Fig. 2, principal correlation analysis (PCoA) of isolated bacterial strains using ISSR pattern showed three clouds: A, B1, and B2. These results were similar and agree with [36] who used PCR-based SSR amplification followed by amplicon size determination to analyze the spread of the pathogens (*Haemophilus influenzae*). Furthermore, the utility of inter-simple sequence repeat PCR (ISSR-PCR) assay in the characterization and elucidation of the phylogenetic relationship between the pathogenic isolates of *Vibrio cholerae* [37]. The application of principal component analysis (PcoA) is the most basic multivariate data reduction statistical technique. The application revealed that two out of five principal components were significant (eigenvalue > 1) and contributed 58.07% of the total variation. Pco1 accounted for 32.33%, and Pco2 accounted for 25.71% of the total variation; also, the same grouping pattern was found in the cluster analysis, indicating that significant variation exists in this study. The separation of Pco1 and Pco2 showed that the five bacterial isolates were dispersed in all quarters, indicating a high level of genotypic variation among the bacterial isolates. These results were parallel to [38] who performed molecular identification and genetic diversity among *Photorhabdus* and *Xenorhabdus* microbes.

### Genetic diversity of selected isolates using SDS-PAGE

**Fig. 3:**
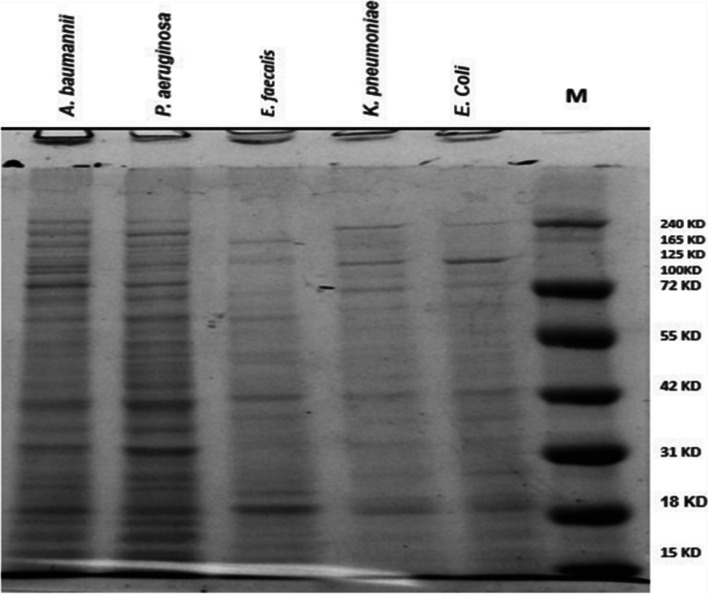
Patterns of SDS-PAGE electrophoretic protein of the selected strains. Lane1: protein ladder (PageRuler™ Plus Prestained). Lane (2–6): represent isolates from (1–5) which represent as *E. coli*, *K. pneumoniae*, *E. faecalis*, *P. aeruginosa*, and *A. baumannii*

SDS-PAGE as an important simple, powerful, and rapid molecular technique for microbial species identification for comparing microbial pathogens [39]. SDS-PAGE analysis showed a total of 66 bands with molecular weight ranging from 10 to 250 KD. Eight polymorphic protein bands at the molecular size of 250, 130.0, 70.0, 35.6, 30.00, 28.00, 18.55, and 10.0 KD. Also, eight monomorphic protein bands as fragment sizes of 100, 75.0, 55.5, 25.0, 20.0, 15.0, 14.0, and 12.0 KD for all tested bacterial isolates (Fig. 3**)**.

**Fig. 4:**
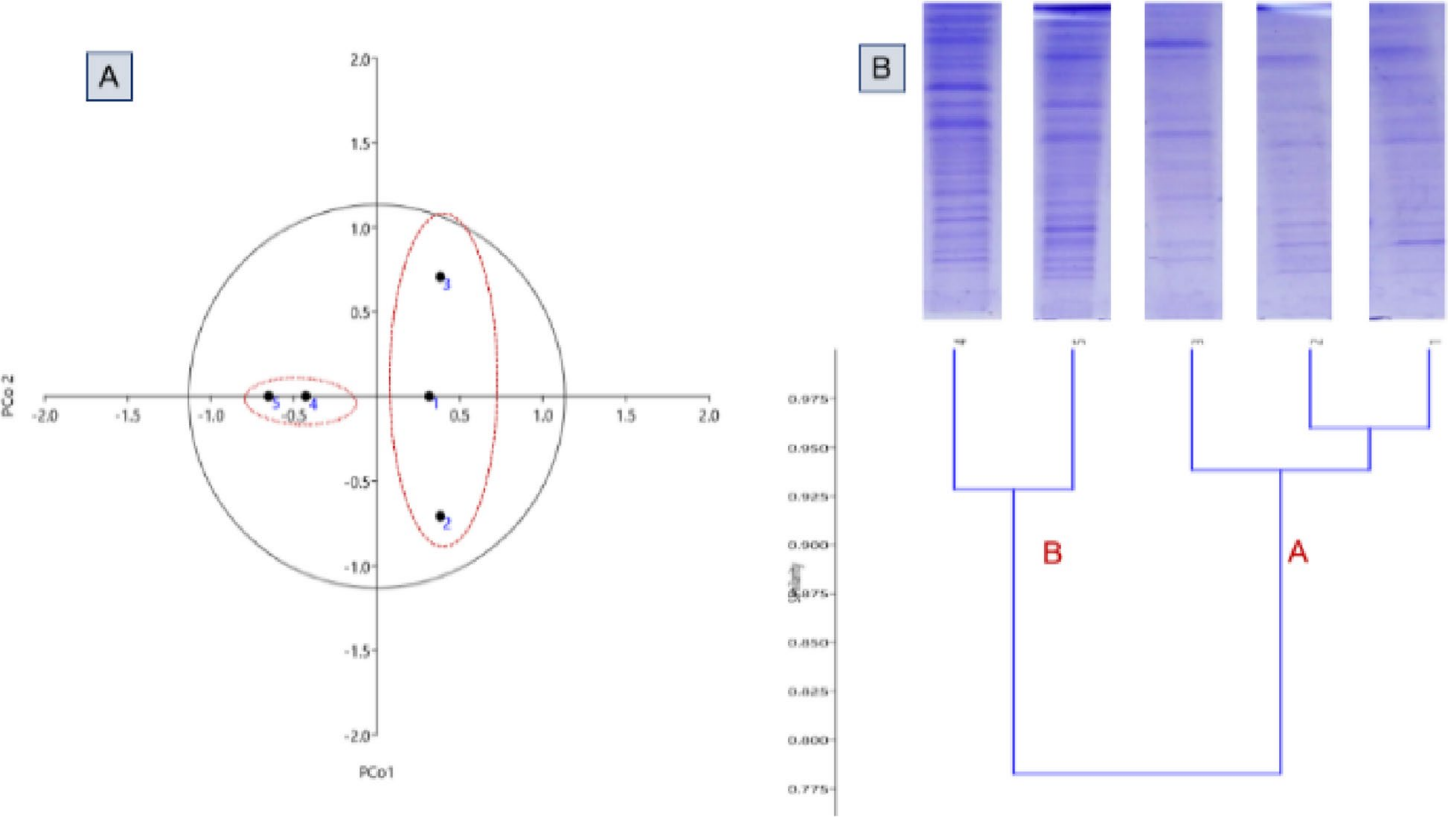
**A** Principal component analysis (PCA) of the selected strains. **B** Dendrogram of SDS-PAGE based on unweighted pair-group method with arithmetic averages algorithm (UPGMA) for the selected strains, 1–5 represent as *E. coli*, *K. pneumoniae*, *E. faecalis*, *P. aeruginosa*, and* A. baumannii*

Data presented in Table 5 explained that polymorphism percentage (%) was 50.0% for all polymorphic bands. Total fragments of all isolates were 66 fragments, and bacterial isolate *E. coli*, *K. pneumoniae*, *E. faecalis*, *P. aeruginosa*, and *A. baumannii* were produced 13, 12, 12, 15, and 13 fragments, respectively. Also, the polymorphism information content (PIC) was calculated as 0.231 generated from the SDS-PAGE pattern. Therefore, our results was similar with [40] reporting that the genetic similarity and protein profile of the isolated field German strains were compared to *Mycoplasma bovis* reference strain using SDS-PAGE. In addition, [39] examined the total proteins of 20 isolates that were taken from the NIH Islamabad using SDS-PAGE to determine the variability in the gene pool of *E. coli*, as shown in Fig. 4. Principal component analysis (PCA), a powerful tool and the fundamental multidimensional data reduction statistical approach, found that two of the five principal component analysis remained significant (eigenvalue > 1) and accounted for 85.86% of the total variance. PC1 accounted for 72.99% of the overall variance, whereas PC2 accounted for 12.87%. When PC1 and PC2 were separated, different microbial strains emerged in all quarters, showing a significant amount of genotypic variety.

**Table 5:** Polymorphism generated by SDS-PAGE of the selected strains

**MW**	*E. coli*	*K. pneumoniae*	*E. faecalis*	*P. aeruginosa*	*A. baumannii*	**Frequency**	**Polymorphism**	**PB**	**MB**	**Unique**	**TB**	**P%**	**PIC**
250.0	0	0	0	1	1	**40.0**	**Polymorphic**	8	8	-	16	50.00	0.231
130.0	1	1	0	1	1	**80.0**	**Polymorphic**						
100.0	1	1	1	1	1	**100**	**Monomorphic**						
75.00	1	1	1	1	1	**100.0**	**Monomorphic**						
70.00	1	1	1	1	0	**80.0**	**Polymorphic**						
55.60	1	1	1	1	1	**100.0**	**Monomorphic**						
35.04	1	1	1	1	0	**80.0**	**Polymorphic**						
30.00	1	1	1	0	0	**60.0**	**Polymorphic**						
28.00	0	0	0	1	1	**40.0**	**Polymorphic**						
25.00	1	1	1	1	1	**100.0**	**Monomorphic**						
20.00	1	1	1	1	1	**100.0**	**Monomorphic**						
18.55	1	0	1	1	1	**80.0**	**Polymorphic**						
15.00	1	1	1	1	1	**100.0**	**Monomorphic**						
14.00	1	1	1	1	1	**100.0**	**Monomorphic**						
12.00	1	1	1	1	1	**100.0**	**Monomorphic**						
10.00	0	0	0	1	1	**40.0**	**Polymorphic**						
Total	**13**	**12**	**12**	**15**	**13**	**66 bands**							

*PPB* number of polymorphic bands, *MB* number of monomorphic bands, *TB* number of total bands, *%P* percent polymorphism, *PIC* polymorphism information content

The numerical analysis clearly revealed two distinct clusters as shown in the dendrogram; it was classified into two main clusters (A and B). Cluster A includes two isolates (*P. aeruginosa* and *A. baumannii*), and cluster B includes three isolates (*E. coli*, *K. pneumoniae*, and *E. faecalis*); these results were similar to [41] who reported that genetic diversity of the dominant bacteria isolated in western Iran. Also, These results were parallel to [42]. They stated that the SDS-PAGE whole cell protein patterns and 16S rRNA gene sequence analysis were used to compare the biodiversity of lactic acid-producing bacteria in garlic and green onion samples. Also Jamalzadeh et al., 2021 [43] said that the SDS-PAGE approach, in combination with computational analysis of protein profiles, is an excellent way for investigating taxonomic relationships among *Bacillus* species. SDS-PAGE was also used to characterize two species of bacteria, *Pseudomonas aeruginosa* and *E. coli* [44].

### Genomic identification of extensive MDR isolates

**Fig. 5:**
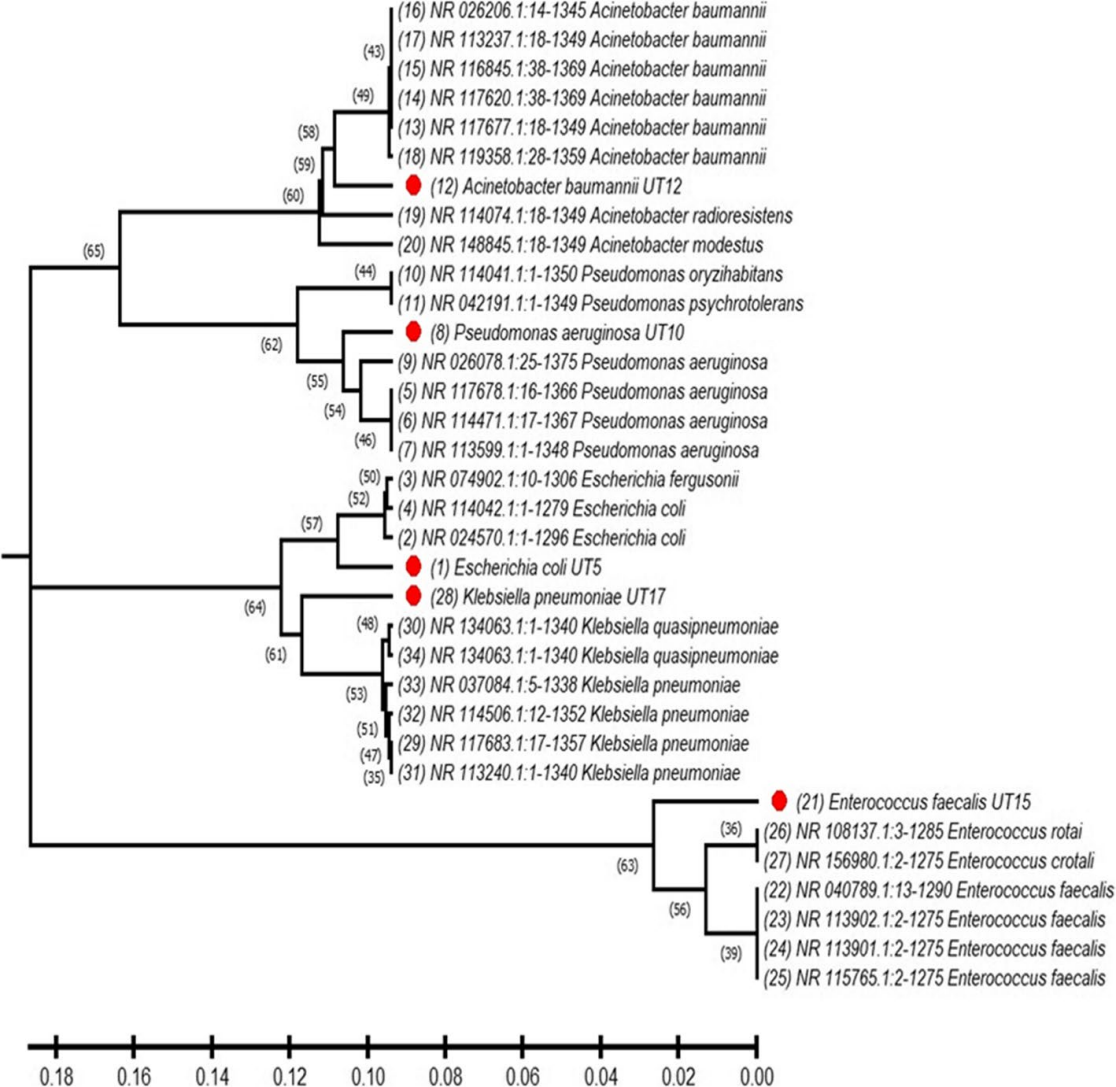
Phylogenetic tree of the extensive five multidrug-resistant bacterial isolates. [Clade A] include A. *baumannii* and other species belong to the Acinetobacter genus. [Clade B] include *P. aeruginosa* and other species belong to the *Pseudomonas* genus. [Clade C] include *E. coli* and other species belong to the Escherichia genus. [Clade D] include *K pneumoniae* and other species belong to the *Klebsiella* genus. [Clade E] include *E. faecalis* and other species belong to the *Enterococcus* genus

The different five extensive MDR bacterial isolates were identified by 16S rDNA gene sequencing and submitted through GenBank database. They were identified as *E. coli*, *P. aeruginosa*, *A. baumannii*, *E. faecalis*, and *K. pneumoniae* with accession numbers OP741103, OP741104, OP741105, OP741106, and OP741107, respectively. Also, phylogenetic trees for the five isolates were also generated in comparison with top ten hints for each one through NCBI BLASTn tool and phylogenetic trees then modified through MEGA11 software as in Fig. 5. Phylogenetic tree of multidrug-resistant bacterial isolates shows the evolutionary relationships between the five clusters of multidrug-resistant bacterial isolates. Clade A includes *A. baumannii*, Clade B includes *P. aeruginosa*, Clade C includes *E. coli*, Clade D includes *K. pneumoniae*, and Clade E includes *E. faecalis*. The tree suggests that these five clusters of bacteria have evolved from different common ancestors and have evolved different mechanisms of antibiotic resistance.

### Characterization of plant-mediated NPs

#### UV–Vis absorption spectra

**Fig. 6:**
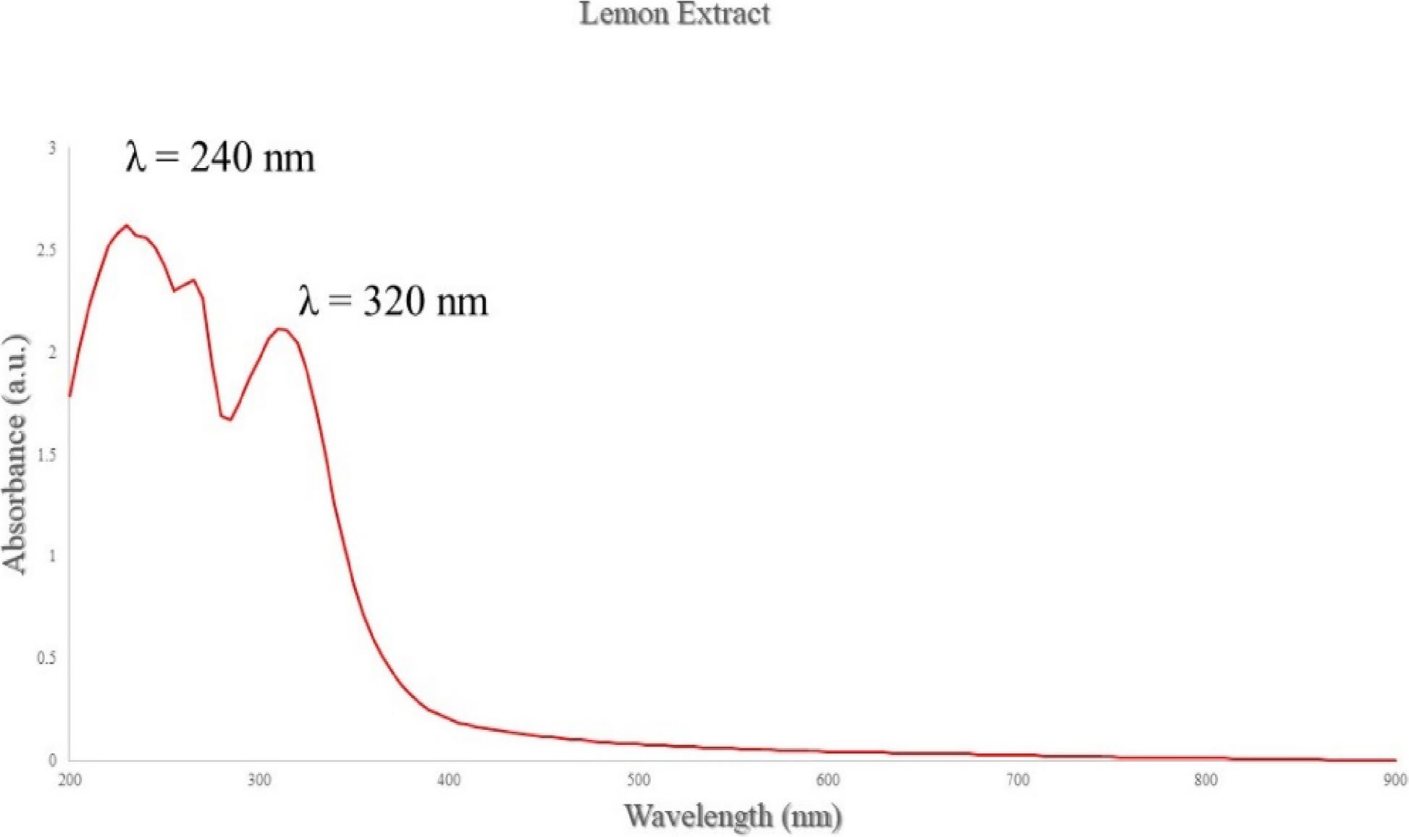
Surface plasmon absorption bands (SPR) of Ag-NPs capped with lemon extract

**Fig. 7:**
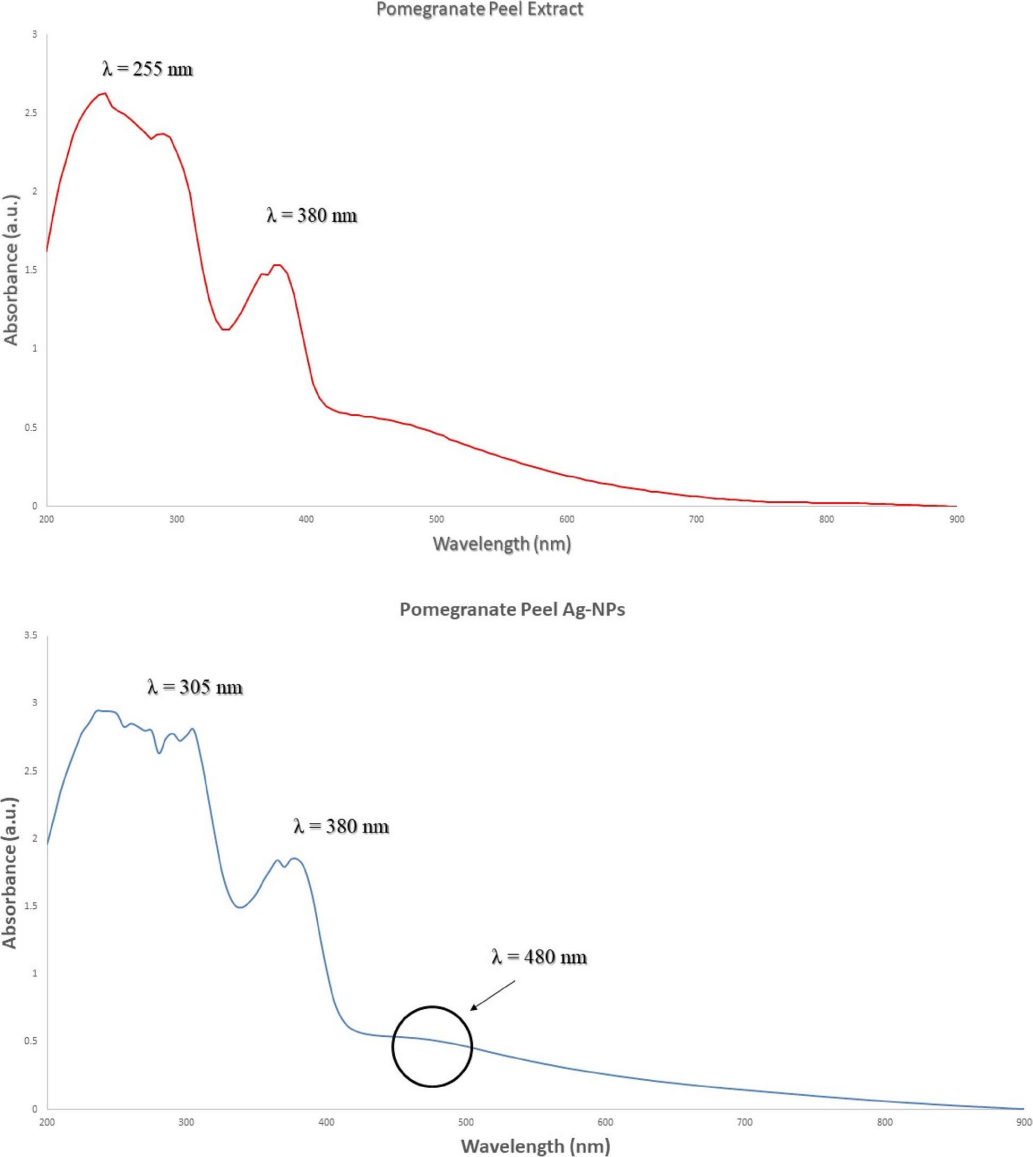
Surface plasmon absorption bands (SPR) of Ag-NPs capped with pomegranate extract

In our study, Ag-NPs were prepared via a green synthesis method. The synthesis was mediated at 32 °C under dark conditions. After the addition of plant extract supernatant, a dark brown color was obtained in the biomass after reaction with the Ag^+^ ions which is confirmed by UV–Vis absorption spectra after 20 min. The as-prepared Ag-NPs which synthesized in the presence of lemon extract exhibit a characteristic surface plasmon bands (SPR) at wavelength 445 nm, indicating to the formation of Ag-NPs (Fig. 6). While Ag-NPs synthesized in the presence of lemon extract exhibit a characteristic surface plasmon bands (SPR) at wavelength 480 nm, indicating to the formation of Ag-NPs (Fig. 7).

#### FTIR analysis

**Fig. 8:**
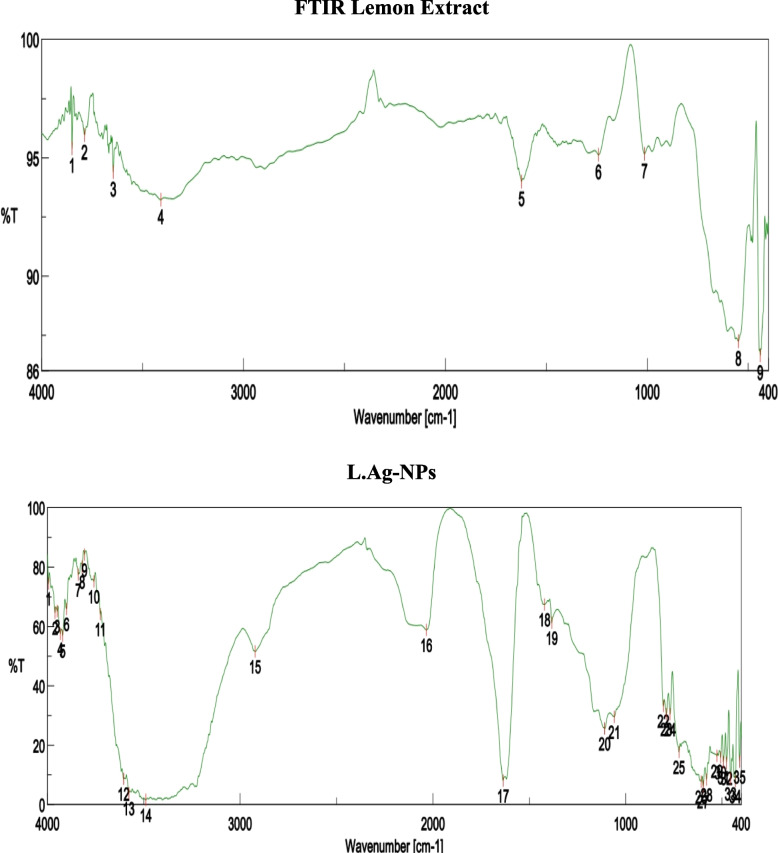
FTIR spectra of lemon extract and L Ag-NPs

**Fig. 9:**
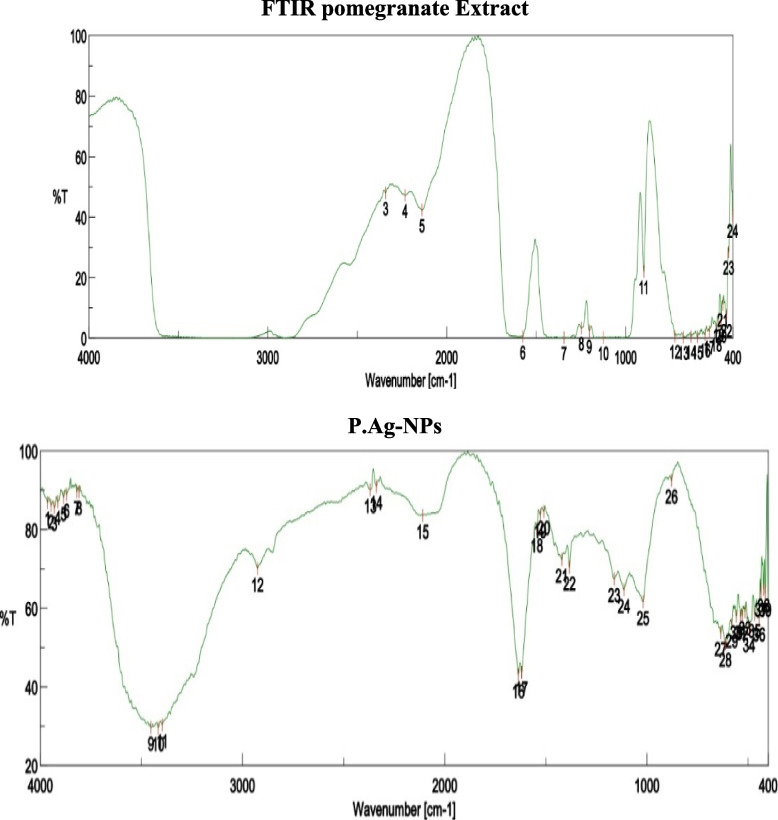
FTIR spectra of pomegranate extract and P.Ag-NPs

Fourier transform infrared (FTIR) spectroscopy studies were used to determine biological molecules necessary for silver nanoparticle reduction, capping, and beside stability. There were many functional groups present that may have been responsible for Ag + ion bio-reduction. The band intensities of plant extracts and silver nanoparticles in various places were investigated. The FTIR spectrum of lemon leaf extract exhibits prominent peak areas at 3409, 1623, 1241, and 548 cm^−1^ (Fig. 8). The presence of plant leaves extract as a capping agent with silver nanoparticles in the sample is shown by the spectra with many marginal changes in peak locations. The peaks at 400–700 cm^−1^ show the presence of inorganic functional groups and may suggest the existence of silver nanoparticles in the sample. The 3409 cm^−1^ peak corresponds to C–H stretching in alkene. The peak at 1623 cm^−1^ belongs to N–O stretching vibrations of the extract’s group, whereas the peak at 1241 cm^−1^ relates to C–N stretching vibrations of the extract’s group. Moreover, the FTIR spectra of pomegranate leaves extract exhibit large peak areas at 3396, 2926, 1383, and 400 to 700 cm^–1^ (Fig. 9). The spectrum from 400 to 700 cm^–1^ shows the presence of Ag-NPs and peaks with some marginal shifts in the region of the peaks indicating the presence of leaf extract as a coating agent in the sample. Peaks located at 400–700 cm^–1^ indicate the presence of inorganic functional groups and may contain silver nanoparticles in the sample. The peak located at 3396 cm^–1^ corresponds to the OH segment in the phenol/carboxylic group. The peak at 2926 cm^–1^ corresponds to the C–H stretching vibrations of the alkene group present in the extract, and a peak at 1383 cm^–1^ can be attributed to the group C–O stretching vibrations present in the extract. In fact, the peaks detected were due to the presence of several secondary metabolites in the leaf extract.

#### XRD analysis

**Fig. 10:**
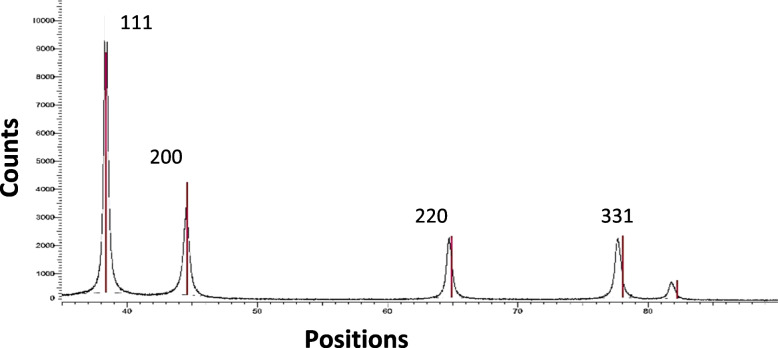
Ray diffraction (XRD) pattern of Ag-NPs from both lemon and pomegranate extracts. Peaks were assigned to diffraction from the (111), (200), and (220) planes of silver. Peaks of XRD pattern can be indexed as a pattern shows the presence of the diffraction peaks corresponding to the (111), (200), (220), and 331 planes

The XRD patterns of Ag-NPs prepared through lemon and pomegranate extracts show 20 values of 38.37°, 44.51°, 64.55°, and 77.86° and weak to moderately intense diffraction peaks at (111), (200), (220), and the (311) plane of metallic Ag with FCC crystal symmetry (Fig. 10). As a result, a higher peak intensity suggests that there are more atoms present in the crystal, which could indicate that the crystal is larger or more crystallin. A lower peak intensity, on the other hand, can indicate a smaller crystal size or a lower level of crystallinity.

#### TEM analysis

**Fig. 11:**
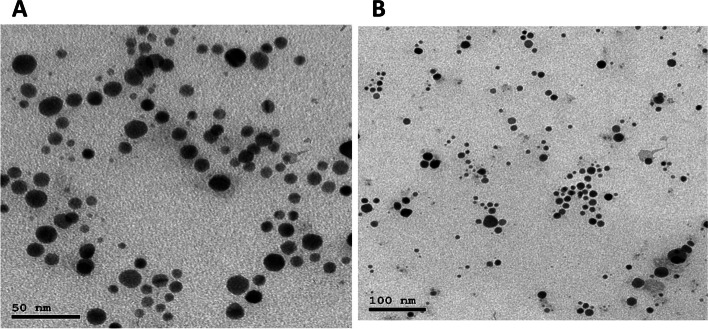
TEM image of AgNPs where **A** L-AgNPs while **B** P-AgNPs

Both Ag-NPs from lemon extract and pomegranate extract were characterized through TEM which exhibits L-Ag-NPs spherical structures with size 32 nm, while P-Ag-NPs are also spherical structures with size 28 nm, with zeta potential − 20.3 and − 25.4 mV for L-Ag-NPs and P-Ag-NPs, respectively (Fig. 11). At greater concentrations, the [Ni-NPs], which have an estimated size of 50 nm, show effective anti-MRSA actions. Compared to other nosocomial bacterial infections, [Ni-NPs] exhibit more potent antibacterial activity against MRS. The UV–Vis spectroscopy, DLS, and TEM analyses of the single and probe-conjugated AuNPs were performed. Using DLS analysis, the diameter of the individual particles was 20.5 nm.

### MDR isolates growth inhibition through plant-synthesized NPs

**Fig. 12:**
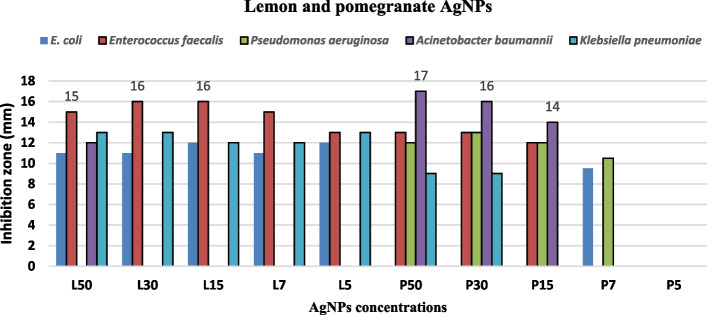
Inhibitory effect of different concentrations of both lemon and pomegranate NPs on the extensive MDR isolates

**Fig. 13:**
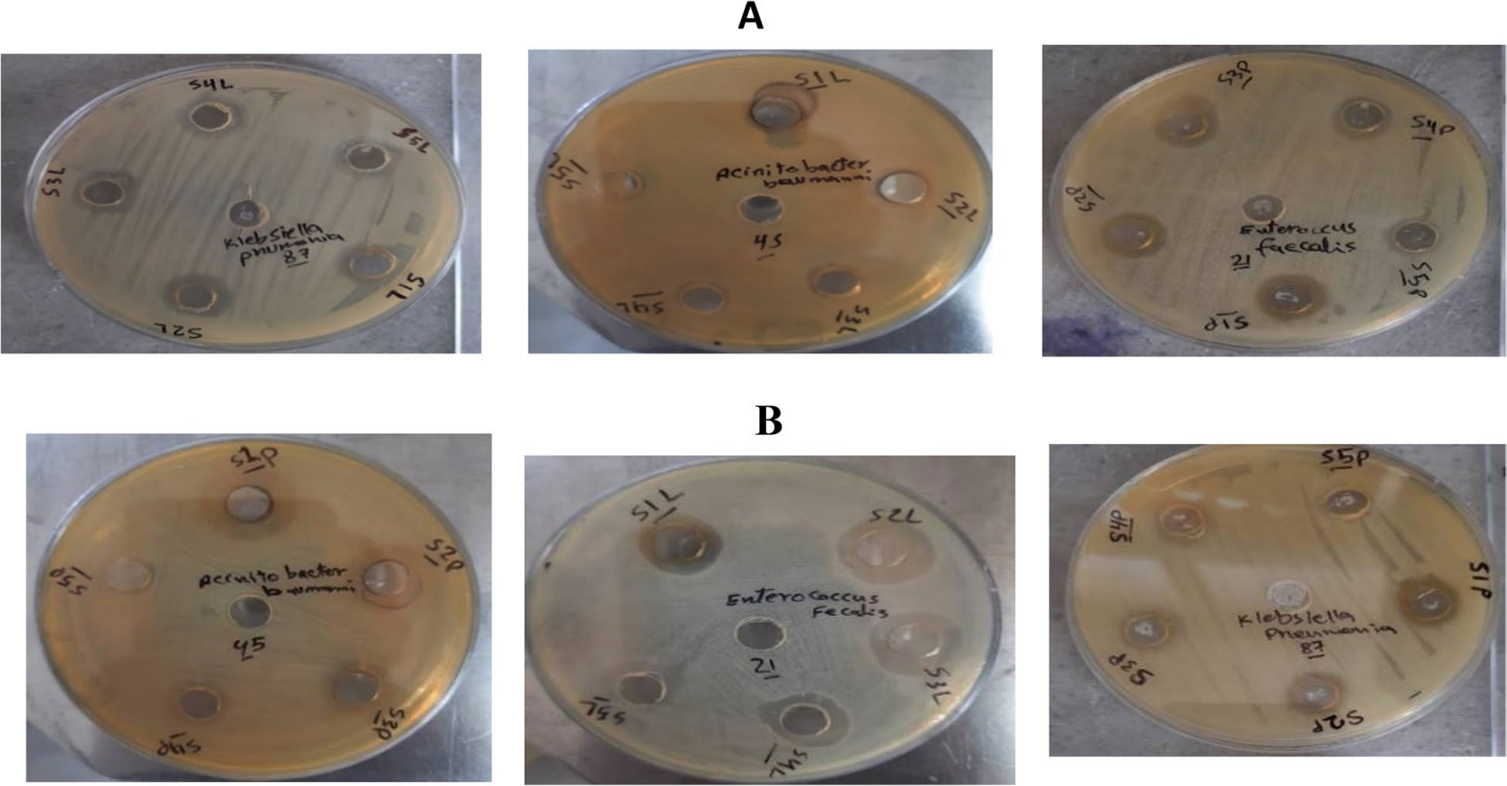
Agar well diffusion assay of plant-mediated NPs on the tested five isolates. **A** Lemon NPs. **B** pomegranate NPs

Lemon extract [L-Ag-NPs] and pomegranate extract [P-Ag-NPs] were utilized as precursors for the synthesis of silver nanoparticles, and both were measured at varied doses of 50, 30, 15, 7, and 5 ug/mL. The MIC for [L-Ag-NPs] was 50 µg/mL, and the MIC for [P-Ag-NPs] was 30 µg/mL. *E. coli*, *E. faecalis*, *A. baumannii*, and *K. pneumoniae* were the four isolates that were inhibited at [L-Ag-NPs] MIC, with inhibition zone widths of 11, 15, 12, and 13 mm, respectively. In addition, four isolates *E. faecalis*, *P. aeruginosa*, *A. baumannii*, and *K. pneumoniae* had [P-Ag-NPs] MICs that inhibited them at 13, 16, 9, and 9 mm, respectively (Figs. 12 and 13).

Ag-NPs prepared through *Moringa oleifera* plant leaves were effective as inhibitory agent at high concentrations 2 mg/mL against *Enterococcus faecalis* OU510, and *Enterococcus faecalis* ATCC29212 as a positive control, and an inhibition zone diameter of 8–10.5 mm or on agar plate. Their MIC was 0.25 mg/mL for both identified bacterial isolates [45].

## Conclusion

The isolated bacterial isolates exhibited MDR activity in about 72% of cases. With the accession numbers OP741103, OP741104, OP741105, OP741106, and OP741107, respectively, the top five MDR bacterial isolates (*Escherichia coli*, *Pseudomonas aeruginosa*, *Acinetobacter baumannii*, *Enterococcus faecalis*, and *Klebsiella pneumoniae*) were determined through 16S rDNA PCR sequencing. ISSR PCR was used to analyze the genetic makeup of these common bacterial isolates. All of the successfully obtained genetic and protein analysis data may serve as a useful tool for understanding and combating the MDR behavior of uropathogens and improving UTI infection treatment strategies. Plant-mediated silver nanoparticles were used to inhibit four different isolates out of five. L-AgNPs, at a concentration of 50 µg/mL, inhibits *E. coli*, *E. faecalis*, *A. baumannii*, and *K. pneumoniae*, while P-AgNPs, at a concentration of 30 µg/mL, inhibits *E. faecalis*, *P. aeruginosa*, and *A. baumannii*. A TEM picture of two [AgNPs] revealed that L-AgNPs were 32 nm in size and P-AgNPs were 28 nm in size.

### Supplementary Information

**Additional file 1.**

**Additional file 2.**

## Data Availability

All data included in this study were presented in the form of tables and figures.
