# Supplementary material for: Green synthesis of silver nanoparticles derived from lemon and pomegranate peel extracts to combat multidrug-resistant bacterial isolates
Source: J Genet Eng Biotechnol. 2023 Oct 10;21:97. doi: 10.1186/s43141-023-00547-0 (PMC10564695; doi:10.1186/s43141-023-00547-0)
Supplement: Supplementary file 2 — Additional file 2. [file 43141_2023_547_MOESM2_ESM.docx]

| **The most effective antibiotics** | | | | |
| --- | --- | --- | --- | --- |
|  |  |  |  |  |
| *SUMMARY* | *Count* | *Sum* | *Average* | *Variance* |
| **Ampicillin** | 77 | 785 | 10.19481 | 18.50103 |
| **Ampicillin/ Sulbactam** | 77 | 774 | 10.05195 | 24.10253 |
| **Piperacillin/ Tazobactam** | 77 | 1330 | 17.27273 | 53.56938 |
| **Cefoxitin** | 77 | 1133 | 14.71429 | 37.6015 |
| **Ceftazidime** | 77 | 1384 | 17.97403 | 39.68353 |
| **Ceftriaxon** | 77 | 1319 | 17.12987 | 50.08817 |
| **Cefepime** | 77 | 1274 | 16.54545 | 60.27751 |
| **Meropenem** | 77 | 1429 | 18.55844 | 56.17088 |
| **Amikacin** | 77 | 1325 | 17.20779 | 26.90362 |
| **Gentamicin** | 77 | 1243 | 16.14286 | 32.25564 |
| **Tobramycin** | 77 | 1121 | 14.55844 | 32.48667 |
| **Ciprofloxacin** | 77 | 1457 | 18.92208 | 58.5728 |
| **Levofloxacin** | 77 | 1549 | 20.11688 | 49.973 |
| **Nitrofurantoin** | 77 | 1188 | 15.42857 | 33.93233 |
| **Trimethoprim/ Sulfamethoxazole** | 77 | 1002 | 13.01299 | 52.93404 |
| **Aztreonam** | 77 | 1157 | 15.02597 | 61.68353 |
| **Ertapenem** | 77 | 1539 | 19.98701 | 55.85509 |
| **Imipenem** | 77 | 1754 | 22.77922 | 36.06904 |

| ANOVA |  |  |  |  |  |  |
| --- | --- | --- | --- | --- | --- | --- |
| *Source of Variation* | *SS* | *df* | *MS* | *F* | *P-value* | *F crit* |
| Rows | 17889.67 | 76 | 235.3904 | 7.338819 | 2.63E-59 | 1.292426 |
| Columns | 14032.21 | 17 | 825.4242 | 25.73443 | 4.68E-70 | 1.630681 |
| Error | 41440.51 | 1292 | 32.0747 |  |  |  |
|  |  |  |  |  |  |  |
| Total | 73362.39 | 1385 |  |  |  |  |
